# Persistent Nodules on Necklines Following Hyaluronic Acid Filler: A Case Report

**DOI:** 10.1111/jocd.70442

**Published:** 2025-09-09

**Authors:** Salma AlBargawi

**Affiliations:** ^1^ Department of Dermatology, College of Medicine Imam Mohammad Bin Saud University Riyadh Saudi Arabia

**Keywords:** dermal injection, filler, hyaluronic acid, necklines

## Abstract

**Background:**

Necklines are a common complaint in patients as they are a sign of aging. Hyaluronic acid (HA) fillers are widely used to address volume loss and linear depressions. HA fillers are safe, effective, and versatile, but their use for necklines is not well‐documented in the literature.

**Aims:**

To report a rare case of persistent, treatment‐resistant early‐onset nodules in the neck follwoing HA "skin booster" filler injection. Furthermore, to highlight the importance of injector expertise as this complication often arises from technical error.

**Patient/Methods:**

A 40‐year‐old woman presented with nodules on the neck following HA "skinbooster" filler (Restylane Vital; Galderma; Sweden). The nodules were firm, non‐tender, non‐inflamed, and located in the superficial layer of the skin, suggesting that a superficial injection of the filler had been performed. She reported undergoing five separate sessions of hyaluronidase, yet no improvement was observed and the nodules persistent for 6 months afterwards.

**Results:**

At 1 week, significant reduction in neck nodules after 300 IU/mL of hyaluronidase was injected. The patient reported high satisfaction with the outcome.

**Conclusions:**

This case highlights the importance of injector expertise and technique, especially when treating critical areas like the neck. Moreover, further research is needed to determine the optimal protocol for persistent and treatment‐resistant complications such as this case.

## 1 Introduction

Necklines are a common complaint, appearing in all age groups despite being a sign of aging [1]. Several treatments have been recommended, including radiofrequency (RF)‐microneedling, high‐intensity focused ultrasound (HIFU), botulinum toxin injections, laser resurfacing, and surgical lifting [1]. Hyaluronic acid (HA) fillers are widely used in aesthetic dermatology to address volume loss and linear depressions. HA fillers are known for their safety, efficacy, and versatility [2], but their use for necklines is not extensively documented in the literature. Filler complications are low in incidence and generally mild, with lumps being the most common complication [2]. Filler complications may be classified by severity, ischemic involvement, and timing of onset [2]. Delayed complications are typically defined as occurring more than 4 weeks to years after treatment [2]. Early lumps are typically painless and often result from improper techniques such as placing the filler too superficially, injecting excess volume, or selecting the wrong product for the specific indication [2]. While complications from HA fillers are typically addressed with the hyaluronidase enzyme, there are limitations to this approach [3]. This case report highlights a particularly challenging presentation of persistent, treatment‐resistant early‐onset nodules in the neck following HA filler injection. The successful management of this rare, neck‐specific complication through a higher than typical concentration of hyaluronidase underscores a critical evidence gap in the literature regarding optimal treatment protocols for such a complication and unique clinical experience.

## 2 Case Description

A 40‐year‐old woman sought treatment for her horizontal necklines at a different clinic where the treating physician performed HA “skinbooster” filler (Restylane Vital; Galderma, Uppsala, Sweden) injections to the area. The patient reported that the nodules appeared immediately after injection and became prominent a few days afterwards. She returned to the clinic in an attempt to dissolve the filler nodules. Despite undergoing five separate sessions of dissolution with hyaluronidase, no improvement was observed. The nodules persisted for 6 months before she sought treatment at our clinic.

**FIGURE 1:**
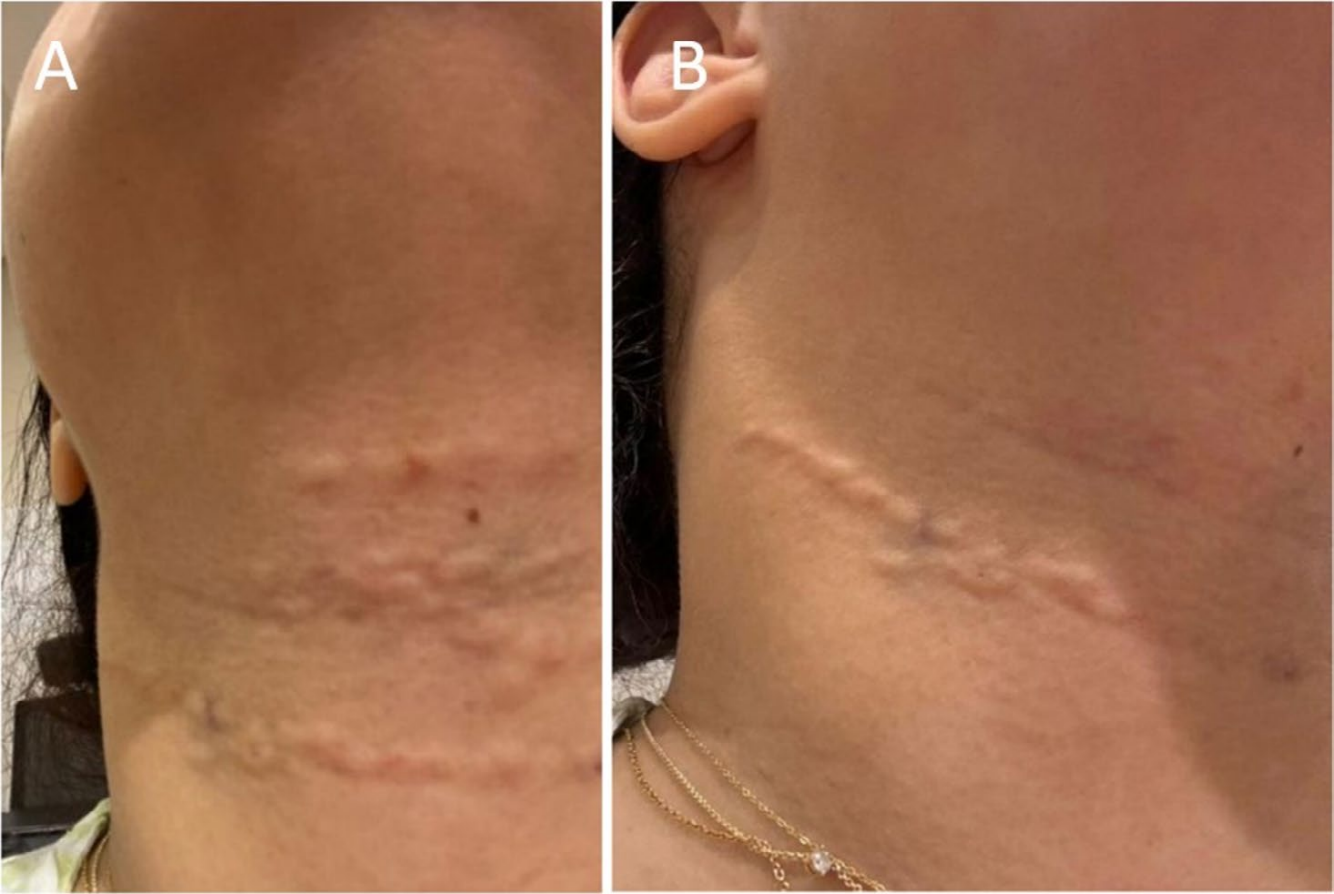
Persistent neck nodules following HA filler treatment, present for 6 months despite five sessions of hyaluronidase. (A) Anterior view of the neck nodules. (B) Anterolateral view of the neck nodules.

Upon assessment, the patient had an unremarkable medical history and had never previously undergone filler treatments. She presented with nodules that were firm, non‐tender, non‐inflamed, and located in the superficial layer of the skin (Figure 1), suggesting that a superficial injection of the filler had been performed. While the superficial injection technique is commonly used to treat necklines, a technical error may have contributed to the development of this complication.

Our working diagnosis was noninflammatory nodules that presented acutely after the injections, likely due to technical error such as too superficial placement and excessive volume, rather than granuloma formation or infection. Clinically, the nodules were clearly palpable and visible in the superficial dermis. No ultrasound imaging was performed at our clinic to confirm the depth of these nodules, as their superficial location was evident upon physical examination.

**FIGURE 2:**
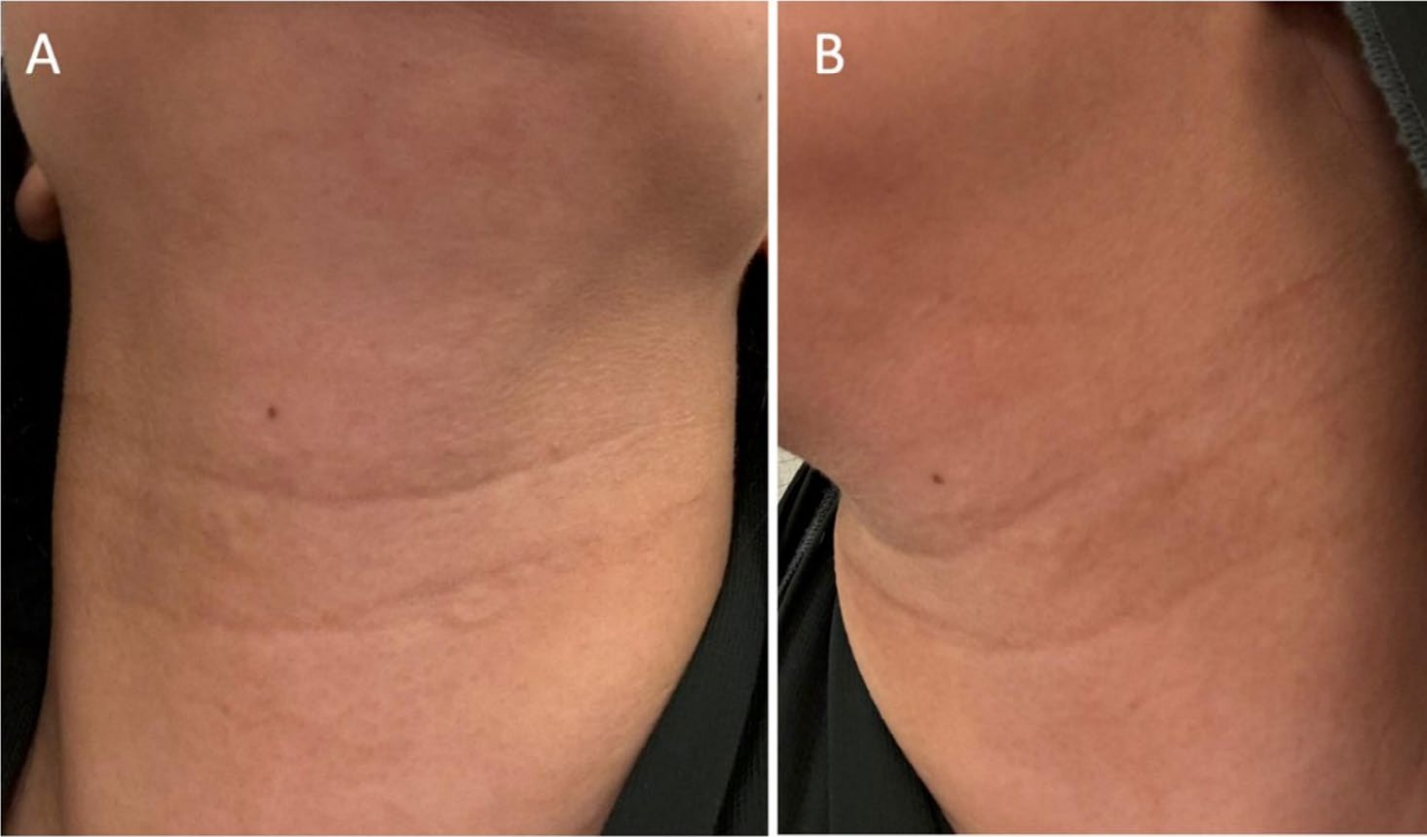
One‐week follow‐up showing significant reduction in neck nodules after 300 IU of hyaluronidase. (A) Anterior view of the neck. (B) Lateral view of the neck.

Given the failure of previous multiple hyaluronidase treatments and the persistence of the nodules, we opted for a higher than typical dose of hyaluronidase, thoroughly injected into the area. A topical anesthetic (lidocaine 2.5% and prilocaine 2.5%; PRILA, Avalon Pharma) was applied to the neck area for 30 min to ensure patient comfort during the injection, as direct injection into nodules can be sensitive. Hyaluronidase (Bovine origin, TOSKANI) was reconstituted with normal saline to 300 international units (IU) per milliliter. Expert consensus recommends 150 IU/mL hyaluronidase for treating persistent noninflammatory nodules [2]. The 300 IU/mL dosage was chosen empirically as a higher concentration, considering the failure of five previous dissolution attempts by the previous clinician with likely standard or lower doses. After cleaning the area, the hyaluronidase was injected through serial puncture superficially and then more deeply within the nodule. In addition, retrograde linear threading injection of the hyaluronidase was done to flood the surrounding area of filler, followed by a gentle massage to ensure even distribution of the enzyme. The patient was instructed to return for a follow‐up in 1 week, to avoid sun exposure in the meantime, and to gently massage the treated area. At follow‐up, clinical examination showed clear improvement with no palpable nodules, no visible edema, and only mild, transient erythema. The patient reported high satisfaction with the outcome (Figure 2).

## 3 Discussion

Studies on HA filler use for necklines have reported positive and sustained outcomes, with side effects being minor and transient [4, 5, 6]. The injection technique described in the literature involves using a 30‐ to 32‐Gauge needle to inject directly into the dermal plane of the horizontal neckline. A retrograde linear threading technique parallel to the skin is done, with the typical injection volume per site ranging from 0.002 to 0.04 cc [4, 5]. A serial puncture method can also be employed if the threading technique does not adequately smooth the lines [4]. Furthermore, it is recommended to massage the area for 10 min postinjection to disperse the product [3].

There is no standard guideline for treating nonemergent filler complications like non‐inflamed nodules. Still, an expert panel consensus recommends treating persistent noninflammatory nodules with needle aspiration, minimal stab wound incision with evacuation, or hyaluronidase at a concentration of 150 IU/mL [2]. Considering that the previous clinician likely used this standard dose or possibly a lower one in all five sessions, a higher concentration may have been necessary for effective treatment.

The present case highlights that for recalcitrant nodules, both a higher concentration of hyaluronidase and an adequate volume to thoroughly flood the area may be crucial for successful dissolution. While a 1‐week follow‐up demonstrated significant improvement, it is acknowledged that mild residual edema could potentially mask very minor persistence. However, the improvement observed clinically, including no palpable nodules or notable edema, suggesting effective dissolution. Long‐term follow‐up beyond 1 week would be beneficial to confirm complete and sustained resolution, though this was beyond the scope of this acute management case report.

Interestingly, an ultrasound‐based study by Schelke et al. [7] found that filler material placed within the superficial musculoaponeurotic system (SMAS) layers may not disperse normally, leading to early nodule formation. Furthermore, all the nodules in their study were located within the fascia; however, they did not include patients with persistent nodules in the neck [7].

Our case, presenting with acutely formed noninflammatory nodules, is distinct from these immune‐mediated late‐onset granulomatous reactions and emphasizes the importance of accurate diagnosis in guiding treatment.

As far as we know, this is the first report to describe persistent neck nodules to this extent. Given the challenges in managing persistent filler nodules when typical interventions fail, future research is needed to explore alternative or synergistic approaches to resolve this issue. Further exploration of the factors contributing to filler‐related complications, such as nodule formation in the neck, and well‐tolerated treatment strategies would be valuable for refining treatment protocols and improving patient outcomes.

## Author Contributions

**Salma AlBargawi:** conceptualization, methodology, investigation, data analysis, writing – original draft, writing – review and editing.

## Ethics Statement

Written informed consent was obtained from the patient of this case presentation and any accompanying images.

## Conflicts of Interest

The author declares no conflicts of interest.

## Data Availability

The data that support the findings of this study are available from the corresponding author upon reasonable request.
